# Factors Influencing the Formation of Nitrous Acid from Photolysis of Particulate Nitrate

**DOI:** 10.1021/acs.jpca.3c03853

**Published:** 2023-10-25

**Authors:** R. Sommariva, M. S. Alam, L. R. Crilley, D. J. Rooney, W. J. Bloss, K. W. Fomba, S. T. Andersen, L. J. Carpenter

**Affiliations:** † School of Geography, Earth and Environmental Science, University of Birmingham, Birmingham B15 2TT, U.K.; ‡ Atmospheric Chemistry Department, Leibniz Institute for Tropospheric Research, Leipzig 04318, Germany; § Wolfson Atmospheric Chemistry Laboratories, Department of Chemistry, University of York, York YO10 5DD, U.K.

## Abstract

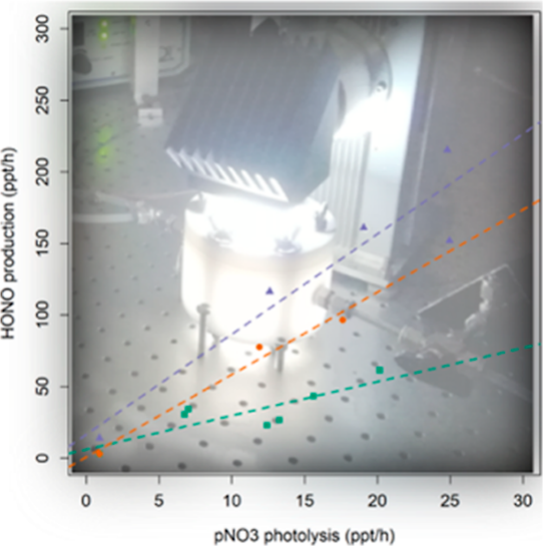

Enhanced photolysis of particulate nitrate (pNO_3_) to form photolabile species, such as gas-phase nitrous acid (HONO), has been proposed as a potential mechanism to recycle nitrogen oxides (NO_*x*_) in the remote boundary layer (“renoxification”). This article presents a series of laboratory experiments aimed at investigating the parameters that control the photolysis of pNO_3_ and the efficiency of HONO production. Filters on which artificial or ambient particles had been sampled were exposed to the light of a solar simulator, and the formation of HONO was monitored under controlled laboratory conditions. The results indicate that the photolysis of pNO_3_ is enhanced, compared to the photolysis of gas-phase HNO_3_, at low pNO_3_ levels, with the enhancement factor reducing at higher pNO_3_ levels. The presence of cations (Na^+^) and halides (Cl^–^) and photosensitive organic compounds (imidazole) also enhance pNO_3_ photolysis, but other organic compounds such as oxalate and succinic acid have the opposite effect. The precise role of humidity in pNO_3_ photolysis remains unclear. While the efficiency of photolysis is enhanced in deliquescent particles compared to dry particles, some of the experimental results suggest that this may not be the case for supersaturated particles. These experiments suggest that both the composition and the humidity of particles control the enhancement of particulate nitrate photolysis, potentially explaining the variability in results among previous laboratory and field studies. HONO observations in the remote marine boundary layer can be explained by a simple box-model that includes the photolysis of pNO_3_, in line with the results presented here, although more experimental work is needed in order to derive a comprehensive parametrization of this process.

## Introduction

Nitrogen oxides (NO_*x*_ = NO + NO_2_) play an important role in the formation of tropospheric ozone and in the atmospheric oxidation capacity.^1−3^ While the levels of NO_*x*_ in urban environments have been thoroughly studied, observations of NO_*x*_ mixing ratios in the <100 ppt range in the marine boundary layer (MBL) remain largely unexplained. The main loss mechanism for NO_*x*_ in the MBL is via the formation of nitric acid (HNO_3_), either by reaction R1 or by nocturnal hydrolysis of N_2_O_5_. Nitric acid is subsequently removed from the gas phase via wet/dry deposition or particle uptake to form particulate nitrate (pNO_3_).
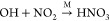
R1

One possible mechanism to recycle NO_*x*_ back into the gas phase is via the photolysis of particulate nitrate (pNO_3_). Laboratory experiments^4−9^ on various substrates have shown that pNO_3_ photolysis (reaction R2) can form gas-phase nitrous acid (HONO) and NO_2_. This process, followed by HONO photolysis to form NO (reaction R3), has been termed “renoxification” and is a potentially important source of NO_*x*_ in the remote boundary layer.
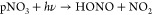
R2
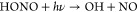
R3

Previous experimental work has shown that “renoxification” chemistry is especially efficient because the photolysis rate of particulate nitrate is enhanced compared to the photolysis of gas-phase HNO_3_. This has been attributed to the pyramidal geometry of HNO_3_, when bound to a surface, which increases its UV absorption cross section.^10^ The result is a faster photolysis rate, although different studies disagree on the value of the enhancement factor (*f*). Reported enhancement factors range from <10 to 1700, depending on the type and composition of aerosol or substrate.^11^ Several parameters are thought to influence the enhancement of pNO_3_ photolysis, which partly explains the wide range of reported *f* values: concentration of NO_3_^–^ ions, pH, humidity, and temperature, solvent cage effects in water droplet or deliquescent particles, presence of other ions and/or organic compounds.^12^

An important consideration in evaluating the importance of this chemistry for the MBL is that some of the experiments reported in the literature were conducted using substrates that are not directly comparable to atmospheric particles, such as urban grime,^4,13^ plant leaves, wood, and metal construction materials.^5^ Other laboratory studies have used bulk aerosol collected on filters,^6,8,14^ with only the work of Shi et al.,^9^ using suspended particles.

Ambient observations of HONO and NO_*x*_ in the remote MBL have provided indirect evidence of “renoxification” chemistry: in the absence of terrestrial sources, and if heterogeneous reactions on the sea surface can be ruled out,^15^ the concentration of HONO can be considered to be controlled only by its photochemistry, which is inadequate to explain the observations, thus suggesting the presence of a HONO source consistent with enhanced photolysis of pNO_3_.^6,7,11,14,16,17^ On the other hand, some ambient studies found that HONO production via reaction R2 is negligible^18^ or can be attributed to an oceanic surface process.^15^

Recent work by Andersen et al.,^11^ proposed a theoretical framework that may explain and bridge the discrepancies in the experimental (laboratory) and ambient studies. They suggest that the pNO_3_ photolysis enhancement factor depends on the partitioning of NO_3_^–^ ions between the bulk and the interface of a deliquesced particle, which can be described by a Langmuir adsorption isotherm. This model broadly fits the reported values of *f* in ambient and artificial substrates, although more studies are clearly needed.

Model investigations support the potential importance of “renoxification” chemistry. Kasibhatla et al.,^19^ for instance, implemented pNO_3_ photolysis in the GEOS-Chem global model, and found that, depending on the value of *f*, this process may result in up to a factor 20 increase in NO_*x*_ and up to a 30% increase in O_3_ in the MBL, especially in tropical and subtropical regions.

This article presents a set of laboratory experiments aimed at exploring the parameter space of pNO_3_ photolysis. Artificial and ambient nitrate-containing particles were collected on filters, and the production of HONO upon illumination of the filters was used to derive nitrate photolysis enhancement factors for a wide range of aerosol compositions and ambient conditions. The experiments presented in this paper focused on the effects of particle composition, (e.g., ammonium sulfate vs sodium chloride), nitrate loading, humidity, and the role of selected organic compounds. The broad aim is to provide a better understanding of how this chemistry contributes to the production of HONO, and hence—extrapolating to ambient conditions—to the atmospheric oxidation capacity.

## Methods

### Experimental Setup

**Figure 1:**
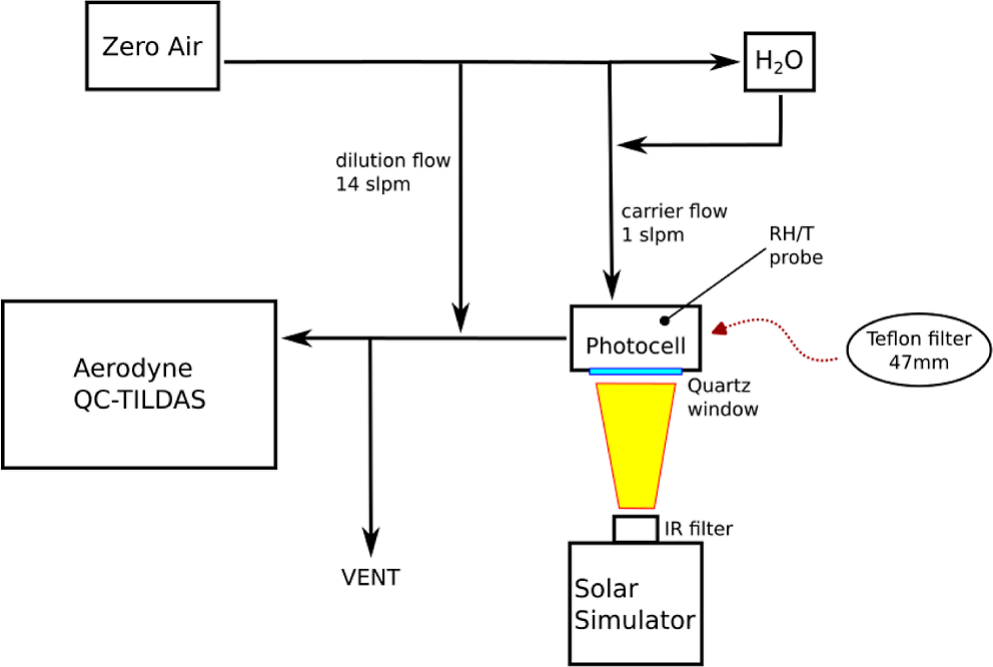
Diagram of the experimental setup.

The experimental apparatus is shown in Figure 1. It consists of a photocell, within which particle samples were illuminated, a solar simulator, and an instrument to measure HONO. Artificial or ambient particles were collected on Teflon filters prior to each experiment, weighed, and placed inside the photocell to photolyze the nitrate contained in the sampled particles and observe the formation of HONO. A zero air generator (Teledyne model T701) was used to provide purified (NO_*x*_ < 0.1 ppb) and dry (RH < 1%) air to the entire experimental apparatus.

The photocell is a block of Teflon machined to the size of the filters (47 mm diameter × 45 mm depth), with two stainless steel connectors to allow zero air in and out. The flow of zero air into the photocell (carrier flow, Figure 1) was kept constant at 1 slpm. A bubbler containing deionized water was used to humidify the carrier flow in some experiments. Because of the high sampling rate of the HONO instrument (14.6 slpm), an additional zero air flow of 14 slpm was added downstream of the photocell (dilution flow, Figure 1). All data were corrected for this dilution. A quartz window (thickness: 2 mm) seals the photocell and allows the light provided by the solar simulator to illuminate the filter—quartz has a transmittance of >90% for the wavelengths emitted by the solar simulator (>280 nm).

The solar simulator (LOT Oriel LS3001) uses a 300 W Xenon lamp (LSB530) with an IR filter to emit light in the UV–visible spectral window between 280 and 700 nm. The output of the solar simulator was determined with a series of actinometric NO_2_ photolysis experiments, described in the Supporting Information (Section S2), and was comparable in intensity to measurements of *j*(NO_2_) in tropical and subtropical locations such as Cape Verde and Delhi, India. A shutter was fitted to the solar simulator to make it possible to block the light without turning off the lamp, thus ensuring a constant output over the duration of an experiment.

### Instrumentation

HONO was measured with a quantum cascade-tunable infrared laser differential absorption spectroscopy instrument (QC-TILDAS, Aerodyne Research Inc.). The instrument was operated according to the manufacturer’s standard operating procedures, with automatic zeroing every hour, giving a precision for HONO of 100 ppt.^20^ The detection limit for HONO during the experiments was determined with a flow of dry zero air as 1.2 ppb (2-σ, 30 s).

Offline ion chromatography (IC) was used to determine the chemical composition of the particles collected on the artificial and ambient filters (see Section S1 in the Supporting Information). The analytical procedure is described in detail in Srivastava et al.^21^ Briefly, a 2 cm^2^ filter punch was placed in a polypropylene tube and extracted with deionized water (10 mL) using a sonication technique for 1 h at 27 °C. The extract was then filtered using a 0.45 μm syringe filter to remove any traces of particles. The extracted filtered solution was stored at 4 °C and analyzed within 14 days of extraction using a high-pressure IC instrument (Dionex Integrion, Thermo Fisher).

### Artificial and Ambient Particles

The experiments were conducted using Teflon filters on which either artificial particles, generated in the laboratory with known composition, or ambient particles had been sampled.

Artificial particles were generated using a TSI 3076 constant output atomizer and collected on 47 mm diameter filters (Pall, PTFE membrane, 2 μm pore size). The output of the atomizer was sampled on a filter for 5 min at a constant flow of 3 slpm. The particle number and size distributions from the atomizer were determined in separate experiments using a scanning mobility particle sizer instrument (SMPS, see Section S1.1 in the Supporting Information), and were stable for a given composition and concentration of the solution used in the atomizer (Figure S1 in the Supporting Information). The artificial particles were generated using a seed of either ammonium sulfate, a major component of inorganic particles, or sodium chloride, a major component of sea-salt particles. A source of nitrate was then added to the solution, either in the form of ammonium nitrate or in the form of sodium nitrate. For some experiments, organic compounds (sodium oxalate, succinic acid, and imidazole) were also added to the solution. For each type of particle, several filters were prepared (Table 1) to assess the variability of individual parameters.

**Table 1:** Summary of the Filter Illumination Experiments^a^

type	numb. filters	particle seed	nitrate source	organics	RH (%)	pNO_3_ (μg)	*f*	*f* uncert. (%)
artificial	7	ammonium sulfate	ammonium nitrate		<1	125–373	2.37	28.5
artificial	1	ammonium sulfate	ammonium nitrate		24	246	4.22	
artificial	1	ammonium sulfate	ammonium nitrate		66	246	2.5	
artificial	2	ammonium sulfate	ammonium nitrate		75–85	245–246	11.76	24.2
artificial	2	ammonium sulfate	sodium nitrate		<1	220–325	5.77	14.9
artificial	2	ammonium sulfate	ammonium nitrate	sodium oxalate	<1	237	4.81	21.3
artificial	2	ammonium sulfate	sodium nitrate	succinic acid	<1	216	4.07	21.4
artificial	2	ammonium sulfate	sodium nitrate	imidazole	<1	236	7.04	17.8
artificial	6	sodium chloride	ammonium nitrate		<1	233–461	7.00	20.5
artificial	1	sodium chloride	ammonium nitrate		<1	2417	1.05	
artificial	1	sodium chloride	ammonium nitrate		<1	3682	0.49	
ambient (Cape Verde)	1	N/A	N/A	N/A	<1	3.5	61.5	
ambient (Cape Verde)	3	N/A	N/A	N/A	<1	13.2–18.6	11.7–16.9	22.7
ambient (Delhi)	2	N/A	N/A	N/A	<1	10151–12384	0.23–0.26	15.4

a The photolysis enhancement factor (*f*) and its uncertainty are the average for each type of experiment (Figure 3). Note that the chemical composition of ambient particles (Cape Verde, Delhi) was not determined.

Ambient particles were collected in Cape Verde, off the west coast of Africa, and in Delhi (India). A MiniVol portable air sampler was used at the Cape Verde Atmospheric Observatory (CVAO, Carpenter et al.,^22^) between November 2019 and February 2020. The MiniVol sampler was fitted with a 2.5 μm impactor (PM2.5) and sampled for 3 days with a flow of 5 slpm, from the top of a 7.5 m tower.

A Thermo Scientific Partisol 2025i-D dichotomous sequential air sampler was used in Delhi, during January–February 2018 to collect fine (PM2.5) and coarse (PM10) particles.^21^ The PM2.5 filters were used for the illumination experiments described in this paper. The Partisol sampler was operated on a 12 h cycle (09:00–21:00 and 21:00–09:00) with a flow of 16.7 slpm. The instrument was located on the campus of the Indian Institute of Technology Delhi (IITD) at 15 m above ground.

Both the MiniVol sampler at CVAO and the Partisol sampler at IITD used 47 mm diameter filters (Pall, PTFE membrane, 2 μm pore size). All filters were weighed before and after sampling to determine the total particle mass collected. The chemical composition of the artificial and ambient particles on the filters, and particularly the concentration of pNO_3_, was determined as described in Section S1 of the Supporting Information.

## Results

In total, 27 artificial aerosol (including blanks, with pNO_3_ = 0 μg) and 6 ambient aerosol (4 from Cape Verde, 2 from Delhi) filter illumination experiments were conducted (Table 1), using the following procedure. In the first part of each experiment, the photocell was sampled empty or with an unused filter inside, in the dark and in the light, to ensure that it was clean and not contaminated, e.g., from previous experiments (the photocell was cleaned with methanol and left under a dry zero air flow between experiments). There were no significant differences between the background signals, determined as explained below, and the signals observed when the photocell was empty nor when it contained an unused filter, which indicates that neither the photocell nor the actual filter can produce measurable HONO in zero air. In the second part of the experiment, a filter on which either artificial or ambient particles had previously been sampled (see the Methods Section) was placed in the photocell.

**Figure 2:**
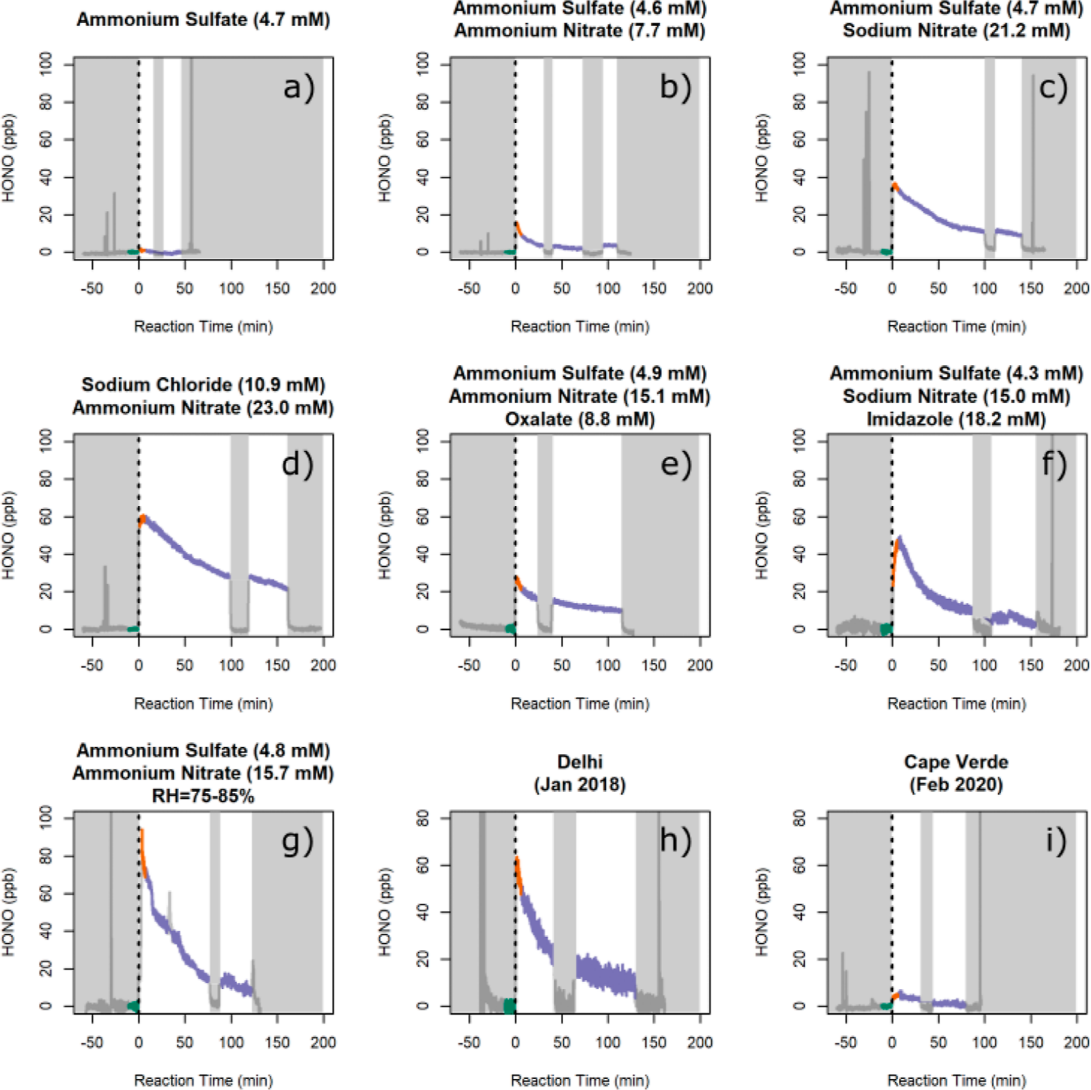
Selected filter illumination experiments. The dotted line indicates when the lamp shutter is first opened; the shaded areas indicate that the shutter is closed. The green lines highlight the interval used to determine the background signal (5 min before the shutter is opened); the orange lines highlight the interval used to calculate *P*(HONO) (5 min after the shutter is opened).

Each filter was first sampled in the dark (lamp shutter closed), to establish the background signal, and then exposed to the light from the solar simulator. The signal during the 5 min before the shutter was opened was used to determine the background HONO signal. The background signal, typically of the order of 2–5 ppb (Figure 2), was then subtracted from the rest of the data. Most of the experiments were conducted in dry zero air and some in humid air (Table 1) to examine whether the production of HONO is affected by humidity.

The results of the experiments are shown in Figure 2 for a selection of artificial and ambient filters. In all experiments, except those with particles that did not contain pNO_3_ (Figure 2a), immediate production of HONO was observed as soon as the shutter was opened for the first time. After the initial spike, HONO production was typically sustained for 1 h or more, decreasing exponentially with time (see below). In all experiments, when the shutter was closed, HONO formation stopped and the HONO signal returned to background levels (Figure 2). HONO formation resumed promptly when the shutter was reopened. Nitric acid was never observed above the detection limit (1.3 ppb) in any of the experiments when the filters were exposed to the light, in agreement with previous work.^9^ The experiments with ambient particles showed patterns of HONO formation similar to those with artificial particles (Figure 2h,2i).

The first 5 min after the shutter was opened for the first time are used hereafter to calculate the production of HONO from the photolysis of pNO_3_. The 5 min interval was chosen because it is long enough to provide a meaningful average and short enough to be representative of the initial HONO production. The HONO production rate, *P*(HONO), was calculated via eq 1
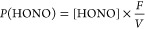
1where [HONO] is the integrated concentration of HONO measured during the first 5 min of exposure to the light (in molecules cm^–3^), *F* is the flow through the photocell (1 slpm), and *V* is the volume of air sampled on the filter (in cm^3^). The calculated *P*(HONO) from eq 1 was then converted to parts per trillion (ppt/h) for ease of comparison with previous studies. The residence time of HONO inside the photocell (volume = 78.1 cm^3^) was only 4.7 s, i.e. 2 orders of magnitude lower than the photolysis lifetime of HONO under the light intensity of the experimental apparatus (435 s, see Section S2 in the Supporting Information). Photolytic loss of HONO inside the photocell can thus be considered negligible under the experimental conditions.

The values of *P*(HONO) varied between 3 and 215 ppt/h for artificial particles and between 10 and 156 ppt/h for ambient particles. These numbers reflect the initial production of HONO when the material deposited on the filters is first exposed to light, and it is apparent that *P*(HONO) decreases with time over the course of an experiment (Figure 2). Typically, the illumination experiments had a duration of 2–4 h. To assess the production of HONO after longer exposure times to the light, the concentration of HONO was estimated by fitting a simple exponential function to the experimental HONO measurements. The extrapolated HONO concentrations indicate that formation of HONO becomes negligible after approximately 4–5 h of exposure to the light.

Some of the filters were weighed after the illumination experiment, which showed that, over a period of 2–4 h, the filters lost on average between 8 and 15% of their mass, depending on the experiment. Assuming that the mass loss was entirely due to photolysis of pNO_3_, it is not enough to explain the observed decrease in HONO production after the initial exposure (Figure 2), which suggests that it is the amount of pNO_3_ available for photolysis that decreases, rather than the total amount of pNO_3_ on the filter. The reason may be that some of the nitrate is not exposed to the light, being deposited on the filter under other material.

## Discussion

The production rate of HONO from the photolysis of particulate nitrate, *P*(HONO), is a function of the amount of pNO_3_ available on the particle and of its photolysis rate, *j*(pNO_3_). As previous experimental work has shown,^6,8,9,14^*j*(pNO_3_) is enhanced by a factor *f* compared to the photolysis rate of gas phase nitric acid, *j*(HNO_3_)
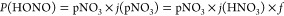
2

**Figure 3:**
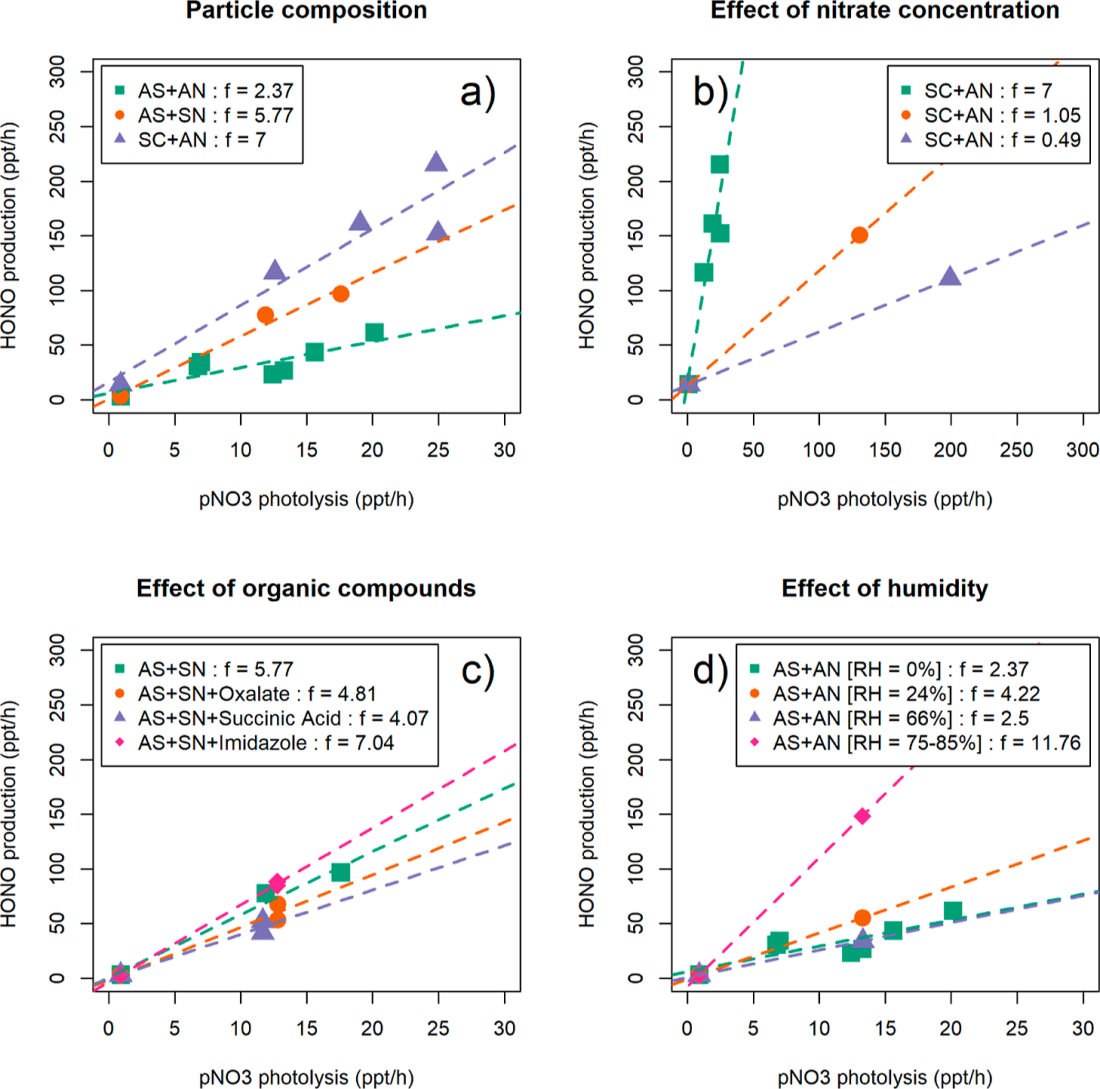
Average particulate nitrate photolysis enhancement factors (*f*) for different types of artificial aerosol. AS = ammonium sulfate, SC = sodium chloride, AN = ammonium nitrate, and SN = sodium nitrate. The concentrations of pNO_3_ in each type of experiments are listed in Table 1.

All terms of eq 2, except for *f*, are known for the experimental system: *P*(HONO) is calculated via eq 1 from the analysis of the experimental data, pNO_3_ is calculated from the concentration of the atomizer solution and/or determined by IC, and *j*(HNO_3_) is determined from *j*(NO_2_) photolysis experiments (see Supporting Information). Therefore, the ratio of *P*(HONO) to pNO_3_ × *j*(HNO_3_) yields the photolysis enhancement factor *f*. Figure 3 shows the *P*(HONO)/pNO_3_ × *j*(HNO_3_) plots for all the experiments with artificial particles, grouped by type of experiment, with their average value of *f*, to highlight the effects of different variables.

The average experimental values of *f* range from 0.49 to 11.76 (Table 1), meaning that particulate phase nitrate can photolyze between a factor of ∼2 slower and a factor of ∼12 faster than gas-phase nitric acid under the experimental conditions. The overall uncertainties in the values of *f* are between 15 and 28% (average = 21%), with the main uncertainty factors being the photolysis rate of HNO_3_ and the concentration of pNO_3_ (eq 2). Both factors are affected by the assumptions in their determination (see Supporting Information), and because of the linearity of eq 2, the uncertainties in *j*(HNO_3_) and/or pNO_3_ propagate proportionally to the value of *f*.

**Figure 4:**
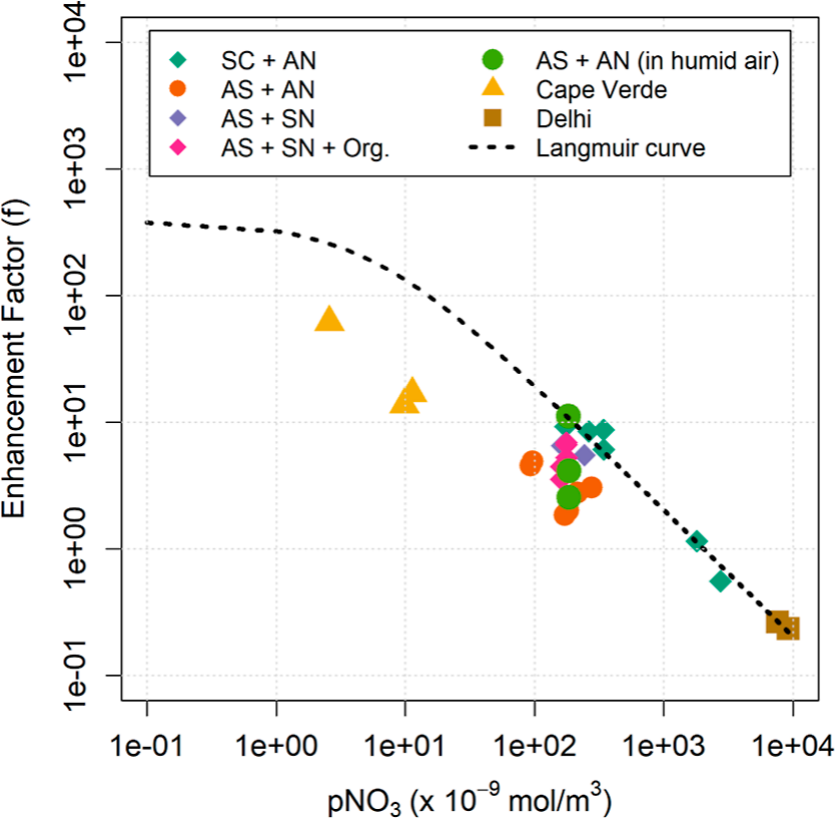
Particulate nitrate photolysis enhancement factors (*f*) for artificial and ambient aerosol. AS = ammonium sulfate, SC = sodium chloride, AN = ammonium nitrate, SN = sodium nitrate. The Langmuir adsorption curve was calculated with the parameters derived from the ambient observations by Andersen et al.^11^

The photolysis enhancement factors in Figure 3 show a clear dependence on the particle composition. In particular, the concentration of nitrate in the particles appears to be the most important variable controlling the value of *f* (in dry air), which is consistent with previous studies.^6,11^ The higher the level of pNO_3_, the lower is the value of *f*, and at pNO_3_ levels above approximately 1 × 10^–6^ mole m^–3^, the photolysis rate of pNO_3_ is the same or slower than that of gas-phase HNO_3_ (Figure 4). The reason for this effect is unclear, and it may be due to surface interactions between NO_3_^–^ ions or, perhaps more likely, to the fact that at very high concentrations, the material on the filter is in crystalline form. The dependence of *f* on the pNO_3_ level sets an upper limit to the impact that this chemistry can have in the troposphere. As proposed by Andersen et al.,^11^ it can also explain why some studies have found limited effect of pNO_3_ photolysis in ambient conditions^7,18^ and low to negligible *f* values in laboratory experiments.^9^

Another important variable that affects the photolysis of pNO_3_ is the presence of cations and halide ions. The former were present as Na^+^ (from sodium chloride, sodium nitrate, or sodium oxalate), and the latter as Cl^–^ (from sodium chloride). Compared with particles containing only ammonium sulfate and ammonium nitrate, the additional presence of sodium resulted in a 2.4-fold higher *f* value (Figure 3). This is consistent with known aqueous phase ion chemistry, whereas Na^+^ ions increase the surface affinity of NO_3_^–^ ions.^23^ The presence of both sodium and chloride additionally increased the value of *f* by 21%: the combined effect of Na^+^ and Cl^–^ has been known to push NO_3_^–^ to the particle interface and to reduce the solvent cage effect.^12,24^ The net result is that more nitrate is available for photolysis, and the yield of pNO_3_ photolysis increases.

Different types of organic compounds were used in these experiments (Table 1): sodium oxalate and succinic acid were chosen because their presence was detected in particle samples at Cape Verde.^25,26^ Imidazole, a known photosensitizer, was chosen in order to compare the results with those of Shi et al.^9^Figure 3 shows that oxalate and succinate suppress the photolysis of pNO_3_ by up to 30%, on average, compared to particles with the same composition but no organics. This may simply be due to the formation of an organic coating on the particles, which prevents light from reaching pNO_3_^–^, or to aqueous phase reactions between the organic compounds and ions or radicals.^12^ On the other hand, the presence of imidazole results in *f* values higher by up to ∼20%, compared to particles without organics. The reason may be that the photosensitivity of some organic compounds can promote aqueous phase reactions;^27^ Wang et al.,^28^ for instance, observed increased formation of nitrite ions (NO_2_^–^) in particles when the photosensitizer vanillic acid was used. However, these results are in disagreement with those by Shi et al.,^9^ who did not observe significant changes in the value of *f* for imidazole-doped particles.

Most of the experiments described above were conducted in dry air (RH < 1%). In some experiments, the carrier flow through the photocell was humidified, to assess the effect of humidity on the photolysis of pNO_3_. For the same initial particle composition, the photolysis enhancement factor increased from 2.37 (RH < 1%) to 4.22 (RH = 24%) to 11.76 (RH = 75–85%), but was lower at intermediate humidity (RH = 66%) with *f* = 2.5 (Figure 3). A possible explanation for this pattern is that at low humidity (below the efflorescence point, RH = 30–35%), water may facilitate photolysis by mobilizing NO_3_^–^ ions on the surface of the filter, and at higher humidity (above the deliquescence point, RH = 70–80%), water promotes the photolysis of pNO_3_ because of increased quantum yield.^12^ Meanwhile, at intermediate humidity, when the particle is in a supersaturated phase, the inhibiting effect of concentrated NO_3_^–^ suppresses photolysis. However, the number of experiments available is small, with only two experiments suggesting that the photolysis of nitrate is less efficient at intermediate humidities, and since it was not possible to establish the phase of the particles deposited on the filters inside the photocell under humid air, this hypothesis should be treated with caution. Previous studies on the effect of humidity on pNO_3_ photolysis have given somewhat contrasting results: for example, Shi et al.,^9^ reported *f* < 10 from sodium nitrate particles at RH > 80%, while Andersen et al.,^11^ observed *f* > 50 from ambient particles at RH > 60%. A study by Baergen and Donaldson^13^ also found a strong dependence of HONO production on humidity, albeit on a very different surface, urban grime. It is clear that more work is required to fully elucidate the role of humidity in pNO_3_ photolysis. The experiments discussed here, which were mostly conducted under dry conditions, should be considered a lower limit for the values of *f* in the troposphere, where humidity is, for the most part, higher than 30% (especially in the MBL).

Figure 4 shows all of the values of *f* determined from both artificial and ambient aerosol as a function of particulate nitrate concentration. The clear trend is that the pNO_3_ photolysis enhancement decreases with increasing pNO_3_, as discussed above. Andersen et al.,^11^ have proposed that the value of *f* in deliquesced particles is dependent on the equilibrium between surface and bulk pNO_3_, and can be explained using a Langmuir adsorption model. The parametrization that they derived from ambient observations overestimates the experimental data in Figure 4, although the dependence on pNO_3_ is broadly similar, especially at higher values. This is not surprising given that most of the experiments were conducted under dry conditions, where particles are not deliquescent. It is worth noting that the *f* values derived from experiments at higher humidities fit the Langmuir curve better than the others.

In terms of nitrate loading, the ambient particles encompass a wide range of conditions, from the clean remote ocean to highly polluted urban location. The ambient particles collected at Cape Verde show *f* values in the range 11.7–61.5, similar to previous studies.^6,7,14,16^ On the other hand, the particles collected in Delhi show very low values of *f* (0.23–0.26), which actually indicate that the photolysis of pNO_3_ is slower than that of gas-phase HNO_3_. The variability in the enhancement factors obtained for ambient particles can be rationalized in view of the results obtained from the artificial particles (see above).

The composition of the Delhi particles is likely highly complex,^21^ with heavy loadings of pNO_3_ and a large number of organic compounds, which suppress pNO_3_ photolysis (Figure 3). High levels of Cl^–^, from industrial and combustion sources are present in Delhi particles, but do not result in enhanced pNO_3_ photolysis due to lack of Na^+^ cations.^24^

Cape Verde particles are mostly constituted of sea salt and therefore rich in Na^+^, Cl^–^ ions and low in pNO_3_. All these factors enhance pNO_3_ photolysis and HONO production (Figure 3). Some of the filters collected in Cape Verde show presence of Saharan dust tracers: high concentrations of K^+^ (>0.25 μg/m^3^), Mg^2+^ (>0.6 μg/m^3^), and Ca^2+^ (>1.2 μg/m^3^), associated with air masses influenced by West Africa—as determined by the FLEXPART back-trajectory model. Dust is known to facilitate the production of HONO via a catalytic mechanism,^29,30^ but the values of *f* derived from these filters are generally lower (<20) than those derived from the filters with no presence of dust tracers (>60). A possible explanation may be that the higher concentration of pNO_3_ overcomes the effect of dust (Figure 4), resulting in an overall lower photolysis enhancement factor.

### Atmospheric Implications

A simple box-model is used here to investigate the impact of enhanced pNO_3_ photolysis on HONO, NO_*x*_, and O_3_ levels under MBL conditions. The chemical mechanism includes only inorganic-CH_4_ chemistry and is taken from the Master Chemical Mechanism v3.3.1 (https://mcm.york.ac.uk/MCM/), with the addition of reaction R2. The model was run, unconstrained, for a period of 24 h using the AtChem2 modeling software.^31^ The initial conditions were set to the average Cape Verde observations during the periods when the ambient filters were collected (see the Methods Section).

The model was run under three scenarios: (a) no pNO_3_ photolysis (base), (b) high pNO_3_ = 8.4 × 10^9^ molecules cm^–3^ (high nitrate), and (c) low pNO_3_ = 1.6 × 10^9^ molecules cm^–3^ (low nitrate). Based on the laboratory experiments discussed above, the photolysis enhancement factors were set to 12 (high nitrate) and 61 (low nitrate), the upper and lower limits derived from particles sampled at Cape Verde (Figure 4).

**Figure 5:**
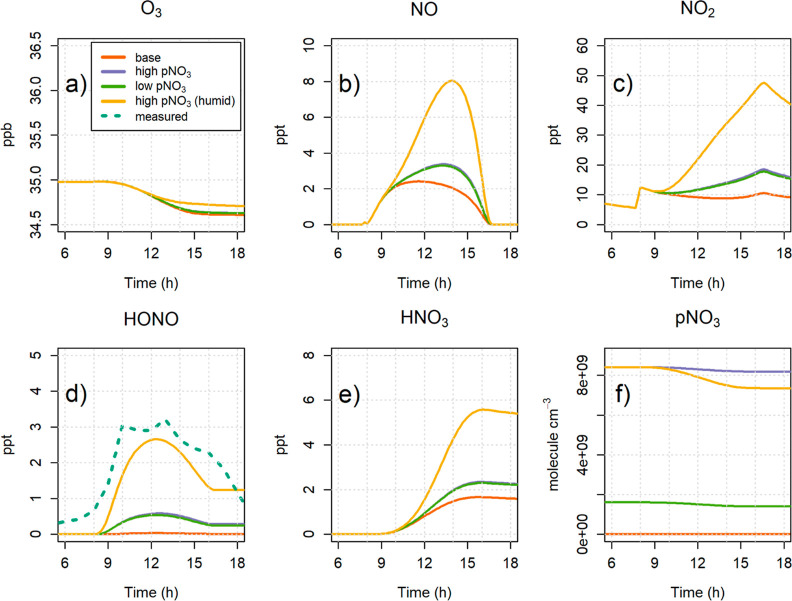
Modeled concentrations of O_3_, NO_*x*_, HONO, HNO_3_, and particulate NO_3_^–^ with different pNO_3_ loadings and photolysis enhancement factors. Measured HONO is the diurnal average of three sets of measurements at CVAO in November 2015, August 2019, and February 2020.

The model results, shown in Figure 5, indicate that there is substantial production of HONO when pNO_3_ photolysis is active, with mixing ratios up to 0.6 ppt produced in the low nitrate scenario. However, average observations of HONO in Cape Verde are of the order of 3–5 ppt.^11,15,16^ The underestimation of HONO production may be explained by considering the effect of the humidity on pNO_3_ photolysis. As discussed above, the photocell experiments conducted in humid air yield higher values of *f*, by up to a factor of 5 for RH > 70% (Figure 3). Indeed, if the high nitrate scenario is run using a photolysis enhancement factor of 60 instead of 12, the model predicts a maximum HONO mixing ratio of about 2.5 ppt, which is in reasonably good agreement with the observations. That being the case, the model shows significant increases in NO and NO_2_ (up to a factor of 4.5) and HNO_3_ (up to a factor of 3.5), in line with previous studies.^16,19^

A few caveats apply. First, the model is highly simplified and contains a very basic chemical mechanism, although it must be noted that previous studies have shown a similarly simple chemical mechanism is able to reproduce the oxidative chemistry in the remote MBL reasonably well.^32^ Second, although the nitrate loading on the filters analyzed in the photocell experiments can be considered representative of ambient conditions (Figure 4), the fact that the particles are deposited on a filter creates an unavoidable difference between what happens inside the photocell and what happens in ambient air on real particles. This difference is hard to characterize and therefore to represent in a model. Finally, it is important to consider that the experiments from which the photolysis enhancement values (*f*) used in the model were derived could only explore a fraction of the wide space of environmental variables involved in this chemistry, particularly in terms of chemical compositions, effects of humidity, and aging of the particles.

## Conclusions

Exposure of artificial and ambient particles to light results in the release of significant amounts of nitrous acid (HONO) due to the enhanced photolysis rate of particulate nitrate (pNO_3_). The efficiency of this chemistry is related to the chemical composition of the particles and to the humidity at which photolysis takes place. The experiments presented in this work cover a wide range of ambient conditions, from very clean marine particles sampled at Cape Verde to highly polluted urban particles sampled in Delhi (India) and a range of artificially generated particles of varying composition. The latter were used to explore the effect of several chemical parameters on the photolysis of pNO_3_. The main findings of these experiments are

- The enhancement of pNO_3_ photolysis, compared to gas-phase HNO_3_, is higher at low pNO_3_ levels, and becomes inhibited at high levels.
- Cations, such as Na^+^, and halides, such as Cl^–^, enhance pNO_3_ photolysis.
- Some organic compounds, such as oxalate and succinic acid, suppress pNO_3_ photolysis, while others which are photosensitive (such as imidazole) enhance it.
- High humidity (i.e., above the deliquescent points of the particles) enhances pNO_3_ photolysis. At lower values, the role of humidity is less clear, although some experiments suggest that pNO_3_ photolysis may be suppressed in supersaturated particles.

Simulations with a simple chemical box-model show that HONO observations in the unpolluted MBL can be reproduced reasonably well by considering the enhancing effects of halides and humidity on the photo-oxidation rate of pNO_3_. However, it must be noted that the laboratory and model results presented in this work should be considered a lower limit for HONO production in the MBL. Real-world particles are very complex, both from a chemical and a physical standpoint, and are suspended in air rather than within a filter matrix.

There is nevertheless strong evidence, from this and other laboratory and field studies reported in the literature, that MBL “renoxification” chemistry via enhanced pNO_3_ photolysis could explain the observations of both NO_*x*_ and HONO in the remote MBL. But there is clearly a need for more experimental information on the impacts of humidity, organic compounds, halides, cations, dust, and other variables on the photolysis of particulate nitrate.

## Supporting Information Available

Information on the characterization of artificial and ambient particles. Determination of the photolysis rates of HONO and HNO_3_ (PDF)
