# Supplementary material for: Factors Influencing the Formation of Nitrous Acid from Photolysis of Particulate Nitrate
Source: J Phys Chem A. 2023 Oct 25;127(44):9302–10. doi: 10.1021/acs.jpca.3c03853 (PMC10641842; doi:10.1021/acs.jpca.3c03853)
Supplement: Supplementary file 1 — jp3c03853_si_001.pdf [file jp3c03853_si_001.pdf]

Factors Influencing the Formation of Nitrous Acid from Photolysis of Particulate Nitrate

R. Sommariva[1], M. S. Alam[1,†], L. R. Crilley[1,‡], D. J. Rooney[1], W. J. Bloss[1], K. W. Fomba[2], S. T. Andersen[3,§], and L. J. Carpenter[3]

[1]School of Geography, Earth and Environmental Science, University of Birmingham, Birmingham, UK

[2]Atmospheric Chemistry Department, Leibniz Institute for Tropospheric Research, Leipzig, Germany

[3]Wolfson Atmospheric Chemistry Laboratories, Department of Chemistry, University of York, York, UK

[†]Current address: School of Biosciences, University of Nottingham, Nottingham, UK

[‡]Current address: WSP Australia, Brisbane, Australia

[§]Current address: Max Planck Institute for Chemistry, Mainz, Germany

Supporting Information

S1 Characterization of particles

S1.1 Artificial particles

Artificial particles were generated from solutions of various composition using a TSI 3076 Constant Output Atomizer (see Methods: Artificial and ambient particles). The number and surface area distributions of the particles were measured

using a TSI 3936 Scanning Mobility Particle Sizer (SMPS). Examples for ammonium sulfate + ammonium nitrate particles are shown in Figure S1.

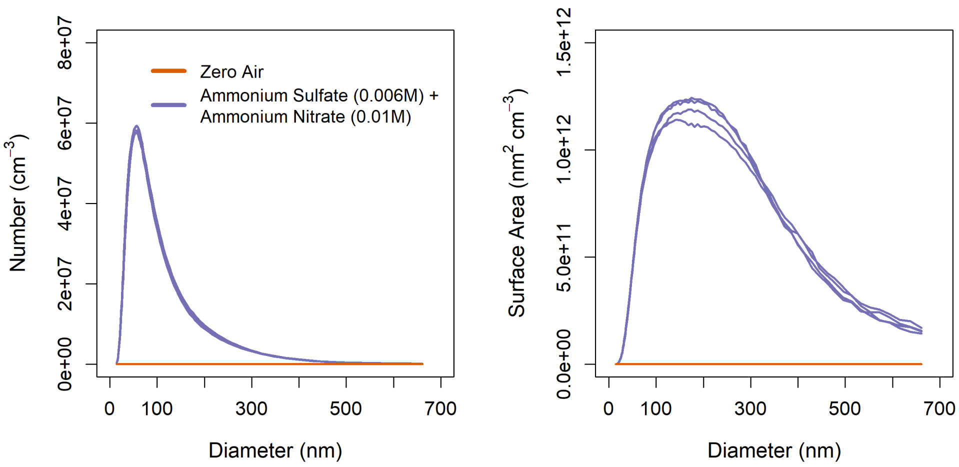

Figure S1: Number and surface area distributions for ammonium sulfate + ammonium nitrate artificial particles.

The chemical composition of the artificial particles is correlated to the composition and concentration of the solution used in the atomizer. The illumination experiments are destructive, meaning that the same filter cannot be both analyzed in the photocell and by ion chromatography. The concentration of particulate nitrate (pNO_3) on the filters was therefore estimated with the following procedure.

First, some of the filters on which artificial particles had been sampled were analyzed by Ion Chromatography (IC, see Methods: Instrumentation), instead of being used for the illumination experiments. Second, the concentration of particulate nitrate, as measured by IC, was found to be linearly correlated to the Molarity of ammonium nitrate or sodium nitrate in the atomizer solution (Figure S2). Third, the correlation between these two parameters was used to estimate pNO_3 from the Molarity of nitrate in the atomizer solution for all the filters that were not analyzed by ion chromatography.

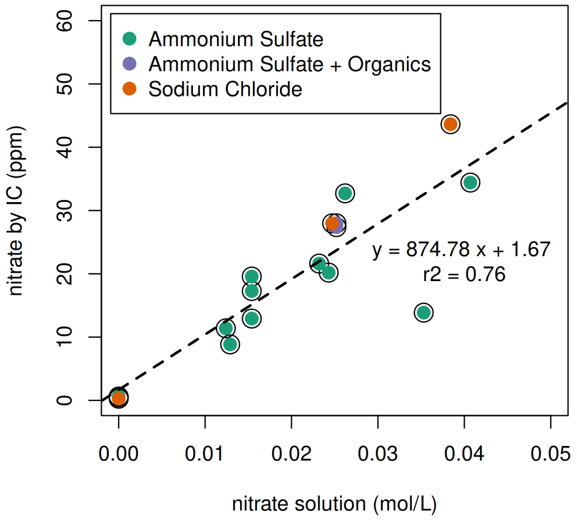

Figure S2: Nitrate concentration on artificial particle filters (measured by IC) vs nitrate concentration in the atomizer solution.

S1.2 Ambient particles

The illumination experiments with ambient particles were conducted with PM2.5 teflon filters collected at Cape Verde and Delhi (India). Since the experimental procedure is destructive, the concentrations of particulate nitrate on the filters that were analyzed in the photocell had to be estimated. In order to do so, contemporaneous and colocated particle composition measurements were used, as described below.

In the case of Cape Verde, filter samples were taken at the Cape Verde Atmospheric Observatory (CVAO, Carpenter et al. (2010)). PM10 samples were collected on quartz filters from the 30 meters tower at CVAO, using a Digitel DHA-80 high volume sampler. To extract the analytes, a quarter of the quartz filter was extracted in deionized water using a sonication technique for 2 hours. The resulting solution was then filtered through a 0.45 μm syringe filter to remove any insoluble particles, and the filtrate was analyzed for standard water soluble ions, including nitrate, using a Dionex ICS3000 ion chromatography system. To account for

background contamination, blank field filters were also analyzed using similar procedures and their concentrations were subtracted from the sample concentrations. For more details on the sampling and analytical procedure, see Fomba et al. (2014); Deabji et al. (2021). PM2.5 samples were collected on teflon filters from the 7.5 meters tower of CVAO: the sampling and analysis of the filters is described in the Methods section. An average PM2.5$_{(7.5m)}$/PM10$_{(30m)}$ ratio of 0.4 for pNO_3 was derived from these measurements and used to estimate the nitrate concentration on the PM2.5 samples that were used in the illumination experiments.

In the case of Delhi, PM2.5 samples were collected using an Envirosense Digitel high volume air sampler (50 slpm) on pre-baked quartz filters (150 mm diameter, Whatman QM-A). The instrument was located on the campus of the Indian Institute of Technology Delhi (IITD), next to the Partisol sampler that collected the PM2.5 samples used in the illumination experiments. The quartz filters were analyzed by ion chromatography, as described in Srivastava et al.. It was assumed that the concentrations of pNO_3 on the quartz and on the teflon filters was the same.

S2 Photolysis rates

The photolysis rate of NO_2 was determined by flowing the photocell with a constant amount of NO_2 and monitoring the mixing ratios of NO and NO_2 when the shutter of the solar simulator was opened (see Methods: Experimental setup). A Thermo Scientific 42i-TL NO-NO_2-NO_x monitor was used for this purpose. The instrument has a stated detection limit of 50 ppt (at 2 minutes averaging time).

Repeat experiments at different levels of NO_2 were made to ensure that the results were reproducible. Two sets of 3 experiments each are shown in Figure S3. The photo-stationary state (PSS) equation was then used to calculate j(NO_2), by fitting Equation S1 to the experimental results:

$$[\mathrm{O_3}] = \frac{\mathrm{j(NO_2)} \times [\mathrm{NO_2}]}{k_{(\mathrm{O_3+NO})} \times [\mathrm{NO}]} \tag{S1}$$

The average from nine NO_2 photolysis experiments yielded a value of 1.33×10^{-2} s^{-1} for j(NO_2), which is comparable to the ambient observations made at Cape Verde (Andersen et al., 2022, 2023).

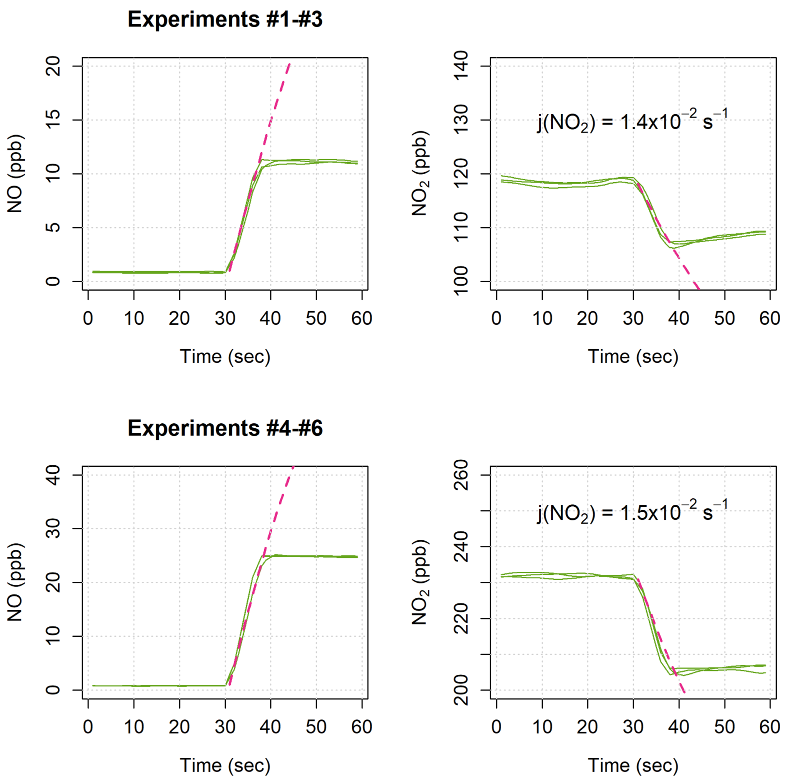

Figure S3: NO_2 photolysis experiments. The red line is the fit to the photo-stationary state equation (PSS, Equation S1).

The photolysis rates of gas phase HNO_3 and HONO are needed for the calculations presented in this paper. These were obtained by calculating the average j(NO_2)/j(HNO_3) and j(NO_2)/j(HONO) ratios, using ambient measurements taken at Cape Verde with a spectral radiometer, as described in the Supplementary Information of Andersen et al. (2022).

Assuming that the same ratios apply to the solar simulator, the average value of j(NO_2) measured in the photocell (1.33×10^{-2} s^{-1}) was scaled, yielding a value of 8.23×10^{-7} s^{-1} for j(HNO_3) and of 2.3×10^{-3} s^{-1} for j(HONO).

References

Carpenter, L. J. et al. Seasonal characteristics of tropical marine boundary layer air measured at the Cape Verde Atmospheric Observatory. *Journal of Atmospheric Chemistry* **2010**, *67*, 87–140.

Fomba, K. W.; Müller, K.; van Pinxteren, D.; Poulain, L.; van Pinxteren, M.; Herrmann, H. Long-term chemical characterization of tropical and marine aerosols at the Cape Verde Atmospheric Observatory (CVAO) from 2007 to 2011. *Atmospheric Chemistry and Physics* **2014**, *14*, 8883–8904.

Deabji, N.; Fomba, K. W.; El Hajjaji, S.; Mellouki, A.; Poulain, L.; Zeppenfeld, S.; Herrmann, H. First insights into northern Africa high-altitude background aerosol chemical composition and source influences. *Atmos. Chem. Phys.* **2021**, *21*, 18147–18174.

Srivastava, D.; Alam, M. S.; Rooney, D. J.; Crilley, L. R.; Kramer, L.; Saksakulkrai, S.; Dhawan, S.; Khare, M.; Shivani; Gadi, R.; Harrison, R. M.; Shi, Z.; Bloss, W. J. The influence of local and regional sources on the composition of particulate matter in Delhi. Submitted.

Andersen, S. T.; Nelson, B. S.; Read, K. A.; Punjabi, S.; Neves, L.; Rowlinson, M. J.; Hopkins, J.; Sherwen, T.; Whalley, L. K.; Lee, J. D.; Carpenter, L. J. Fundamental oxidation processes in the remote marine atmosphere investigated using the NO-NO_2-O_3 photostationary state. *Atmospheric Chemistry and Physics* **2022**, *22*, 15747–15765.

Andersen, S. T. et al. Extensive field evidence for the release of HONO from the photolysis of nitrate aerosols. *Science Advances* **2023**, *9*, eadd6266.
